# Supplementary material for: Convalescent plasma use in the USA was inversely correlated with COVID-19 mortality
Source: eLife. 2021 Jun 4;10:e69866. doi: 10.7554/eLife.69866 (PMC8205484; doi:10.7554/eLife.69866)
Supplement: Supplementary file 1. [file elife-69866-supp1.docx]

**Supplementary File 1**. Mortality of COVID-19 in USA CCP efficacy studies.

| **Study** | **Location** | **Type** | **Mortality** | | **Relative risk of death with plasma** | **Reference** |
| --- | --- | --- | --- | --- | --- | --- |
|  |  |  | CCP | Control |  |  |
| Salazar et al. | Houston, TX | Matched Cohort Design | 6.2 | 12.5 | 0.50 | (Salazar et al., 2021) |
| Liu et al. | New York City | Matched Cohort Design | 12.8 | 24.4 | 0.52 | (Liu et al., 2020) |
| Shenoy et al. | Washington, DC | Matched Cohort Design | 25 | 27 | 0.93 | (Shenoy, Hettinger, Fernandez, Blumenthal, & Baez, 2021) |
| Donato et al. | New Jersey | Matched Cohort Design | 11.1 | 27.1 | 0.41 | (Donato et al., 2021) |
| Yoon et al. | New York City | Matched Cohort Design | 32.8 | 37.5 | 0.87 | (Yoon et al., 2021) |
| O’Donnell et al. | New York City | Double Blind Randomized Controlled | 12.6 | 24.6 | 0.51 | (O’Donnell et al., 2021) |
| Sostin et al. | Hartford, CT | Matched Cohort Design | 20 | 24.6 | 0.81 | (Sostin et al., 2021) |
| Sturek et al. | Charlottesville, VA | Matched Cohort Design | 6.9 | 10.4 | 0.66 | (Sturek et al., 2021) |
| Average |  |  | 15.9 | 23.5 | 0.68 |  |
