# Supplementary material for: Convalescent plasma use in the USA was inversely correlated with COVID-19 mortality
Source: eLife. 2021 Jun 4;10:e69866. doi: 10.7554/eLife.69866 (PMC8205484; doi:10.7554/eLife.69866)
Supplement: Supplementary file 2. [file elife-69866-supp2.docx]

Supplementary file 2. Data from and calculations for excess mortality from CCP hesitancy based on the CDC database.

| **Starting date for seven-day period for Hospitalizations** | **Starting date for seven-day period for deaths** | **Plasma Distributions** | **Deaths** | **Hospitalizations** | **Plasma Doses Per Patient** | **Mortality (Deaths / Admissions - 2 Week Shift)** |
| --- | --- | --- | --- | --- | --- | --- |
| 2020-08-03 | 2020-08-17 | 10511 | 6854 | 31448 | 0.3342343 | 0.2179471 |
| 2020-08-10 | 2020-08-24 | 10495 | 6395 | 29914 | 0.3508391 | 0.2137795 |
| 2020-08-17 | 2020-08-31 | 9218 | 5894 | 28841 | 0.3196144 | 0.2043618 |
| 2020-08-24 | 2020-09-07 | 8968 | 5154 | 28335 | 0.3164990 | 0.1818952 |
| 2020-08-31 | 2020-09-14 | 8424 | 5316 | 25850 | 0.3258801 | 0.2056480 |
| 2020-09-07 | 2020-09-21 | 7894 | 5301 | 24432 | 0.3231009 | 0.2169695 |
| 2020-09-14 | 2020-09-28 | 9647 | 4854 | 22795 | 0.4232068 | 0.2129414 |
| 2020-09-21 | 2020-10-05 | 11670 | 4905 | 23851 | 0.4892877 | 0.2056518 |
| 2020-09-28 | 2020-10-12 | 11101 | 4895 | 26365 | 0.4210506 | 0.1856628 |
| 2020-10-05 | 2020-10-19 | 14796 | 5592 | 32198 | 0.4595316 | 0.1736754 |
| 2020-10-12 | 2020-10-26 | 16401 | 5775 | 35493 | 0.4620911 | 0.1627081 |
| 2020-10-19 | 2020-11-02 | 17827 | 6651 | 39595 | 0.4502336 | 0.1679758 |
| 2020-10-26 | 2020-11-09 | 18628 | 8482 | 45067 | 0.4133401 | 0.1882087 |
| 2020-11-02 | 2020-11-16 | 19205 | 10447 | 54855 | 0.3501048 | 0.1904475 |
| 2020-11-09 | 2020-11-23 | 26176 | 10158 | 69243 | 0.3780310 | 0.1467007 |
| 2020-11-16 | 2020-11-30 | 28076 | 15177 | 80036 | 0.3507921 | 0.1896272 |
| 2020-11-23 | 2020-12-07 | 28688 | 17031 | 84937 | 0.3377562 | 0.2005133 |
| 2020-11-30 | 2020-12-14 | 27350 | 18634 | 92813 | 0.2946785 | 0.2007693 |
| 2020-12-07 | 2020-12-21 | 25706 | 15526 | 99678 | 0.2578904 | 0.1557616 |
| 2020-12-14 | 2020-12-28 | 29842 | 18425 | 102606 | 0.2908407 | 0.1795704 |
| 2020-12-21 | 2021-01-04 | 24350 | 22605 | 103921 | 0.2343126 | 0.2175210 |
| 2020-12-28 | 2021-01-11 | 25475 | 23334 | 109965 | 0.2316646 | 0.2121948 |
| 2021-01-04 | 2021-01-18 | 30576 | 21711 | 114279 | 0.2675557 | 0.1899824 |
| 2021-01-11 | 2021-01-25 | 28946 | 21978 | 106434 | 0.2719620 | 0.2064942 |
| 2021-01-18 | 2021-02-01 | 25395 | 20351 | 92383 | 0.2748882 | 0.2202894 |
| 2021-01-25 | 2021-02-08 | 20226 | 17991 | 79549 | 0.2542584 | 0.2261625 |
| 2021-02-01 | 2021-02-15 | 18913 | 13455 | 67999 | 0.2781364 | 0.1978706 |
| 2021-02-08 | 2021-02-22 | 12303 | 13553 | 54321 | 0.2264870 | 0.2494984 |
| 2021-02-15 | 2021-03-01 | 8369 | 11938 | 45664 | 0.1832735 | 0.2614313 |
| 2021-02-22 | 2021-03-08 | 8429 | 8152 | 40635 | 0.2074320 | 0.2006152 |
