# Supplementary material for: Convalescent plasma use in the USA was inversely correlated with COVID-19 mortality
Source: eLife. 2021 Jun 4;10:e69866. doi: 10.7554/eLife.69866 (PMC8205484; doi:10.7554/eLife.69866)
Supplement: Supplementary file 3. [file elife-69866-supp3.docx]

**Supplementary file 3.** Excess death calculations based on OWID database.

|  | Scenario 1 | | | Scenario 2 | | | Scenario 3 | | |
| --- | --- | --- | --- | --- | --- | --- | --- | --- | --- |
| Transfused | 42.59% | | | 50% | | | 0% | | |
| **Week** | **Expected Deaths** | **Difference** | **Expected Deaths** | | **Difference** | **Expected Deaths** | | **Difference** |  |
| 8/8/2020 | 6048.24 | 1372.76 | 5652.48 | | 1768.52 | 8322.97 | | -901.97 |  |
| 8/15/2020 | 6922.10 | 203.90 | 6469.15 | | 656.85 | 9525.48 | | -2399.48 |  |
| 8/22/2020 | 5999.39 | 711.61 | 5606.82 | | 1104.18 | 8255.75 | | -1544.75 |  |
| 8/29/2020 | 5706.66 | 602.34 | 5333.25 | | 975.75 | 7852.92 | | -1543.92 |  |
| 9/5/2020 | 5469.47 | 385.53 | 5111.58 | | 743.42 | 7526.53 | | -1671.53 |  |
| 9/12/2020 | 5388.78 | -263.78 | 5036.17 | | 88.83 | 7415.49 | | -2290.49 |  |
| 9/19/2020 | 4887.81 | 460.19 | 4567.97 | | 780.03 | 6726.10 | | -1378.10 |  |
| 9/26/2020 | 5130.24 | 308.76 | 4794.54 | | 644.46 | 7059.71 | | -1620.71 |  |
| 10/3/2020 | 4329.12 | 609.88 | 4045.84 | | 893.16 | 5957.29 | | -1018.29 |  |
| 10/10/2020 | 4499.73 | 673.27 | 4205.29 | | 967.71 | 6192.06 | | -1019.06 |  |
| 10/17/2020 | 4988.94 | 233.06 | 4662.49 | | 559.51 | 6865.27 | | -1643.27 |  |
| 10/24/2020 | 6089.31 | -276.31 | 5690.86 | | 122.14 | 8379.49 | | -2566.49 |  |
| 10/31/2020 | 6700.83 | -688.83 | 6262.36 | | -250.36 | 9221.00 | | -3209.00 |  |
| 11/7/2020 | 7408.06 | -105.06 | 6923.31 | | 379.69 | 10194.21 | | -2891.21 |  |
| 11/14/2020 | 8410.37 | -253.37 | 7860.04 | | 296.96 | 11573.49 | | -3416.49 |  |
| 11/21/2020 | 10293.77 | 825.23 | 9620.20 | | 1498.80 | 14165.24 | | -3046.24 |  |
| 11/28/2020 | 12850.57 | -2034.57 | 12009.69 | | -1193.69 | 17683.64 | | -6867.64 |  |
| 12/5/2020 | 14807.61 | 1229.39 | 13838.68 | | 2198.32 | 20376.72 | | -4339.72 |  |
| 12/12/2020 | 15717.11 | 2189.89 | 14688.66 | | 3218.34 | 21628.28 | | -3721.28 |  |
| 12/19/2020 | 17115.82 | 2131.18 | 15995.85 | | 3251.15 | 23553.04 | | -4306.04 |  |
| 12/26/2020 | 18391.33 | -2023.33 | 17187.89 | | -819.89 | 25308.26 | | -8940.26 |  |
| 1/9/2021 | 18996.70 | -90.70 | 17753.65 | | 1152.35 | 26141.30 | | -7235.30 |  |
| 1/16/2021 | 19240.22 | 3867.78 | 17981.23 | | 5126.77 | 26476.41 | | -3368.41 |  |
| 1/23/2021 | 20325.75 | 3440.25 | 18995.74 | | 4770.26 | 27970.22 | | -4204.22 |  |
| 1/30/2021 | 21057.77 | 848.23 | 19679.85 | | 2226.15 | 28977.54 | | -7071.54 |  |
| 2/6/2021 | 19676.24 | 2531.76 | 18388.72 | | 3819.28 | 27076.42 | | -4868.42 |  |
| 2/13/2021 | 17004.92 | 3732.08 | 15892.20 | | 4844.80 | 23400.42 | | -2663.42 |  |
| 2/20/2021 | 14583.63 | 2833.37 | 13629.35 | | 3787.65 | 20068.50 | | -2651.50 |  |
| 2/27/2021 | 12499.40 | 728.60 | 11681.50 | | 1546.50 | 17200.39 | | -3972.39 |  |
| 3/6/2021 | 10062.92 | 3717.08 | 9404.45 | | 4375.55 | 13847.55 | | -67.55 |  |
|  |  |  |  | |  |  | |  |  |
| **Excess Deaths^1^** |  | **37,363.18** |  | | **60,294.31** |  | | **94,436.69** |  |

^1^Excess deaths (difference column) was calculated by subtracting observed deaths in OWID database minus expected deaths according to the linear regression model. The reason the last column in the table includes many negative numbers is because in the absence of plasma increased deaths would have been expected resulting in 94,436 additional deaths.
